# Survey of Women Physicians' Experience with Elected Leadership Positions

**DOI:** 10.1089/heq.2018.0101

**Published:** 2019-04-26

**Authors:** Sasha K. Shillcutt, Sareh Parangi, Sarah Diekman, Reem Ghalib, Robin Schoenthaler, Linda M. Girgis, Ranna Parekh, Hansa Bhargava, Julie K. Silver

**Affiliations:** ^1^ Department of Anesthesiology, University of Nebraska Medical Center, Omaha, Nebraska.; ^2^ Department of Surgery, Harvard Medical School, Boston, Massachusetts.; ^3^ Department of Surgery, Massachusetts General Hospital, Boston, Massachusetts.; ^4^ College of Law, Florida Agricultural and Mechanical University, Orlando, Florida.; ^5^ Texas Clinical Research Institute, Arlington, Texas.; ^6^ Department of Radiation Oncology, Massachusetts General Hospital, Boston, Massachusetts.; ^7^ Department of Family Medicine, Rutgers Robert Wood Johnson Medical School, Piscataway, New Jersey.; ^8^ American Psychiatric Association, Washington, DC.; ^9^ Morehouse School of Medicine, Atlanta, Georgia.; ^10^ Department of Pediatrics, Children's Healthcare of Atlanta, Atlanta, Georgia.; ^11^ Department of Physical Medicine and Rehabilitation, Harvard Medical School, Boston, Massachusetts.; ^12^ Brigham and Women's Hospital, Boston, Massachusetts.; ^13^ Department of Physical Medicine and Rehabilitation, Spaulding Rehabilitation Hospital, Charlestown, Massachusetts.

**Keywords:** women in medicine, gender equity, women physicians, leadership, elections

## Abstract

**Purpose:** Women physicians do not advance in academic promotion or leadership at the same rate as their male counterparts. One factor contributing to academic promotion and advancement is the experience of serving in elected leadership positions. Although >400 women are running for political office in 2018, fewer than a handful are physicians and there has never been a woman physician elected to the Congress. Yet, little is known about women physicians who run for elected positions within their institutions, medical/professional societies, or government. This study sought to examine how women physicians experience elections using a cross-sectional survey of women physicians to gain insight into patterns of reported experiences and perceived barriers to elected leadership positions.

**Methods:** A cross-sectional survey study of 1221 women physicians.

**Results:** 43.8% (N=535) of women physicians ran for an elected office from high school through medical school graduation, in contrast to only 16.7% (N=204) after graduating from medical school. Only 8.5% of women physicians surveyed reported a boss or supervisor encouraged them to run for an elected position.

**Conclusion:** Women physicians are less likely to run for elected positions and for those with previous election experience, the most common barriers cited were lack of institutional time and support, experience, and mentorship.

## Introduction

Numerous studies have documented the disparity between men and women in leadership roles in medicine, particularly at the highest levels.^1–11^ For example, Schor found that only 15% of U.S. medical school deans were women, and the positions they held tended to focus more on image and education as opposed to men who were more likely to focus on corporate strategy and policy, finance, or government relations.^12^ She concluded that gender stereotypes continue to drive the dearth and roles of women in these positions. Helitzer et al. reported that women in academic medicine were not advancing at the expected rate according to *critical mass theory*, which asserts that once the proportion of women faculty reaches a threshold of ∼30%, their impact on the culture of academic medicine should be evident.^13^ After following 1273 faculty at 24 U.S. medical schools for 17 years, Carr et al. recently reported that “women were less likely than men to achieve the rank of full professor” (odds ratio [OR]=0.57; 95% confidence interval [CI]=0.43–0.78).^14^ After the authors adjusted for publications, they found that while “differences by gender in retention and attainment of senior rank were no longer significant…male faculty were more likely to hold senior leadership positions” (OR=0.49; 95% CI=0.35–0.69).^13^ Because women were less likely to advance into senior-level leadership roles, even after adjusting for publication-related productivity, the researchers called on institutions to examine the climate for women to enable them to more fully realize their academic capital and to ensure there is equal opportunity for leadership.

Holding an elected position at one's institution or medical organization is an important step along the path to academic promotion and career advancement for physicians. Elected positions offer the establishment of a national and international reputation, a key requirement for promotion. How elected positions contribute to the advancement of women in medicine is not well described, and there is little information available about women's experiences with elections. To our knowledge, this is the first study in the literature to evaluate trends and patterns of women physicians running for office.

In this cross-sectional survey, we aimed to increase understanding of women physicians' experiences with elections, including whether demographic factors (e.g., ethnicity/race, practice setting, specialty, and children) influenced their experience with and interest in running for elected office. Beyond demographic factors, we also evaluated previous experiences with elections to identify factors that may influence one's willingness to run in the future. For example, we questioned whether a previous electoral success might positively impact a woman's willingness to run again, or if they were unsuccessful, whether that may adversely affect their interest in running for elected office in the future. Finally, we wanted to learn more about what women physicians perceive are the barriers to elected office (e.g., lack of experience and reputational risk) and what they think may help overcome these barriers (e.g., protected time to run and financial support).

## Methods

### Study design

The Institutional Review Board at the University of Nebraska Medical Center approved this study. A cross-sectional survey was posted in two online Facebook groups of women physicians: the Physician Mom Group (PMG; *N*=60,000) and Style M.D. (SMD; *N*=7265). The software utilized was SurveyMonkey^®^ (San Mateo, CA), and each participant was asked to complete the survey only once. The survey was posted in each group for a total of 28 days, from April 26, 2018 to May 24, 2018. The survey collected data on participants' demographics (age, race, ethnicity, and medicine specialty) and questioned participants' opinions about, experience with, and external support for running for elected positions, in addition to perceived personal barriers in running for office. The questions were designed to evaluate trends in running for elected offices during both medical training (undergraduate and medical school) and later as practicing physicians (Table 1).

**Table 1.** Survey Questions of Women Physician Participants

Election history and outcomes
(1) Have you ran for an elected office during your school (high school thru medical school) years?
(2) If yes, did you win?
(3) Since you finished your medical training have you ever run for an elected office at your workplace?
(4) If you ran for an elected office at work, did you win?
(5) Since you finished your medical training have you ever run for an elected office at medical or other professional society?
(6) If you ran for an elected office at a medical or professional society, did you win?
(7) Since you finished your medical training have you ever run for an elected political/government office (local, regional, national)?
(8) If you ran for an elected political/government office, did you win?
Reasons to run for elected positions
(1) In the past, have you considered running for an elective position?
(2) In the past, has anyone encouraged you to run for office?
(3) Who has encouraged you to run for office?
(4) Did the person/people who encouraged you to run in the past offer specific support that would help you to be successful in your candidacy?
(5) What else would encourage you to run for office?
Barriers to running for elected positions
(1) When considering whether you will run for an elected position in the future, what PERSONAL BARRIERS do you think you will face for an elective position?
(2) When considering whether you will run for an elected position in the future, what OTHER BARRIERS do you think you will face?
Women physicians in elected positions
(1) It is important to have women physicians in elected offices, because they provide value in improving health care delivery.
(2) It is important to have women physicians in elected offices in medical and/or professional societies, because they provide value to the organization.
(3) It is important to have women physicians in elected government/political offices, because they provide a more balanced dialogue over important policy issues.
(4) More women physicians should be in elected positions at work.
(5) More women physicians should be in elected positions in medical and/or professional societies.
(6) More women physicians should be in elected positions in the government.
Future possibility of running for elected positions
(1) In the future, I would consider running for an elected office at work.
(2) In the future, I would consider running for an elected office at a medical or professional society.
(3) In the future, I would consider running for an elected political office.
(4) Learning more about disparities in compensation, promotion and other problems for women physicians has encouraged me to consider running for office in the future.
(5) Learning more about how patients are suffering and the need for policy changes has encouraged me to consider running for office in the future.
(6) Watching the current people in office do their jobs ineffectively has encouraged me to consider running for office in the future.
(7) If I was connected to a team of highly skilled individuals who offered me both training and financial support, I would consider running for political office in the future.

### Statistical analysis

We used descriptive statistics to evaluate whether demographic factors (e.g., ethnicity/race, practice setting, specialty, and children) influenced the participant's experience and interest in running for elected positions. We also used descriptive statistics to evaluate women physicians' perceptions of barriers to running for elected offices and how to overcome them.

### Previous experience with elections

Beyond demographic factors, we hypothesized that previous experience with elections may influence a woman physician's willingness to run in the future. Estimated ORs and 95% CIs were used to ascertain if previous experience with elections predicted a woman physician's willingness to run for elected office in the future.

### Perceptions of barriers and how to overcome them

We also used descriptive statistics to evaluate perceived barriers to running for elected offices and how to overcome them. Further analysis to identify barriers and possible solutions for future interventions was carried out on all survey respondents to see if a respondent would consider running for office in the future. In other words, we wanted to know the barriers faced by the respondents who said they would consider running in the future.

## Results

A total of 1221 women completed the survey for analysis; 898 women from the PMG (*n*=900/71,000, 1.3% response rate) and 320 women from the SMD group (*n*=320/7265, 4.4% response rate). The majority of participants were in the 30–39 age group (52.3%), followed by the 40–49 age group. Nearly all the participants identified themselves as being an attending physician (94%) and 44.5% were in academic practice. Demographics of the participants are listed in Table 2 and included women physicians from all over the world, with the vast majority from North America.

**Table 2.** Demographics of Women Physician Participants (Female Gender, *N*=100%)

Survey participants	*N*=1221, *n* (%)
Age, years	
20–29	8 (0.7)
30–39	639 (52.3)
40–49	478 (39.1)
50–59	80 (6.5)
60–69	12 (1.0)
70 plus	2 (0.2)
No response	2 (0.2)
Ethnicity	
Hispanic	83 (6.8)
Race	
Non-Hispanic White or Euro-American	910 (74.5)
Asian, Pacific Islander	208 (17.0)
Black, Afro-Caribbean, or African American	54 (4.4)
South Asian or Indian American	17 (1.4)
Native American or Alaskan	8 (0.7)
Other/no response	8 (0.7)
Middle Eastern or Arab American	7 (0.6)
East Asian or Asian American	5 (0.4)
Latino or Hispanic American	4 (0.3)
Level of medical training	
Attending/practicing physician	1148 (94.0)
Nonpracticing physician	19 (1.6)
Fellow	26 (2.1)
Resident	26 (2.1)
Medical student	1 (0.1)
Practice setting	
University/academic	543 (44.5)
Private practice	379 (31.0)
Other	235 (19.2)
Self-employed	30 (2.5)
N/A	8 (0.8)
Primary medical specialty	
Internal medicine	270 (22.1)
Other (ethics, genetics, hospice, etc.)	19 (15.6)
Pediatrics	159 (13)
Family medicine	152 (12.4)
Obstetrics and gynecology	104 (8.5)
Surgery	82 (6.7)
Anesthesiology	74 (6)
Emergency medicine	74 (6)
Psychiatry	64 (5.2)
Physical medicine and rehabilitation	37 (3)
Neurology	35 (2.9)
Radiology	35 (2.9)
Critical care medicine	21 (1.7)
Pathology	21 (1.7)
Dermatology	13 (1)
Ophthalmology	19 (1.6)
Pathology	21 (1.7)
Working status	
Full time	1014 (83)
Part time	118 (15.4)
Currently not working/no response	19 (1.5)
Relationship status	
Married	1129 (92.4)
In a committed relationship	45 (3.7)
Single/other/no response	48 (3.9)
Children	
1 or more school-aged children	1062 (87)
Adult child(ren) only older than 18 years	63 (5.2)
No children	61 (5)
1 or more nonschool-aged children	34 (2.8)
No response	1 (0.1)

### Previous experience with elections

The survey found that 43.8% (*n*=535) of women physicians ran for an elected office from high school through medical school graduation, in contrast to only 16.7% (*n*=204) after graduating from medical school. A total of 38.8% of women physicians reported winning an election from their high school through medical school years, whereas 12% reported winning after graduating from medical school (Table 3). Although 43.2% (*n*=527) of the women reported having considered running for elected office, a minority of respondents (29.5%) reported they were encouraged to run by another person, and only 8.5% said they had a boss or supervisor encourage them to run for an elected position. Of the women who previously ran for elected office at their workplace since completion of their medical training, age was found to be associated with running for elected positions, whereas race, specialty, or having children (either of school age or nonschool age) were not. A test for trend association between running for office and reported age demonstrated that as the age group of the respondents increased, so did the percent running for elected positions (*p*<0.001).

**Table 3.** Survey Responses from Women Physicians on Elections

	Ran for office, % (*n*)	Won an election, % (*n*)
Election history and outcomes		
High school, medical school, residency and fellowship	43.8 (535)	38.8 (474)
Postmedical training	16.7 (204)	12.1 (148)
Ran for office at workplace	16.7 (204)	12.1 (148)
Ran for office at medical/professional society	13.5 (165)	9.9 (121)
Ran for government office (local, regional, national)	0.9 (11)	0.7 (8)
Experience with running for elected positions		
Considered running for an elected position in the past	43.2 (527)	
Has been encouraged to run for elected position in the past	29.5 (360)	
Boss/supervisor encouraged her to run for office	8.3 (101)	
Beliefs on women physicians in elected positions	Agree/strongly agree	
It is important to have women physicians in elected offices because		
They provide value in improving health care delivery	95 (1161)	
They provide value to the organization	96.9 (1183)	
They provide a more balanced dialogue over important policy	94.5 (1154)	
More women physicians should be in elected positions in		
Workplace	95.4 (1165)	
Medical and/or professional societies	96.3 (1176)	
Government	95.9 (1170)	
Future possibility of running for elected positions	Agree/strongly agree	
In the future, I would consider running for an elected office at		
Workplace	55.1 (672)	
Medical and/or professional societies	49.6 (606)	
Government	17.8 (217)	
Perceived barriers to running for elected positions in the future		
Lost time from family	87.1 (1063)	
Lack of experience	57.5 (702)	
Uncomfortable with self-promotion	50.6 (618)	
Lack of mentorship	43.8 (535)	

### Perceived barriers and how to overcome them

The most commonly reported internal barriers were lack of experience (57.6%), being uncomfortable with self-promotion (50.6%), and not a good use of time (31.3%). The most common external barriers were lack of time/work support (87.1%), lack of experience or training in running for office (57.5%), and lack of mentorship (43.8%). Of interest, only 18.8% of women reported lack of qualifications as a reason not to run for elected office, whereas nearly a third (28.7%) responded they did not think others would consider them to be qualified for the job (Table 3).

The majority of women physicians surveyed agreed or strongly agreed that having women in elected offices would improve health care delivery (95%) and bring value to health care societies and national organizations (96.9%). The majority (58.7%) also reported they would consider running for an elected office in the future at their workplace, and 49.6% said they would consider running for an elected office in their medical or professional society.

## Discussion

This survey revealed that women are less likely to run for elected positions after finishing medical training than before completion, despite the fact that the majority of respondents thought there should be more women physicians in elected positions. Second, although a large percentage of women physicians surveyed (43.2%) had considered running for an elected position, fewer than 10% were encouraged to do so by a boss or supervisor.

### Previous experience with elections

**FIG. 1.**
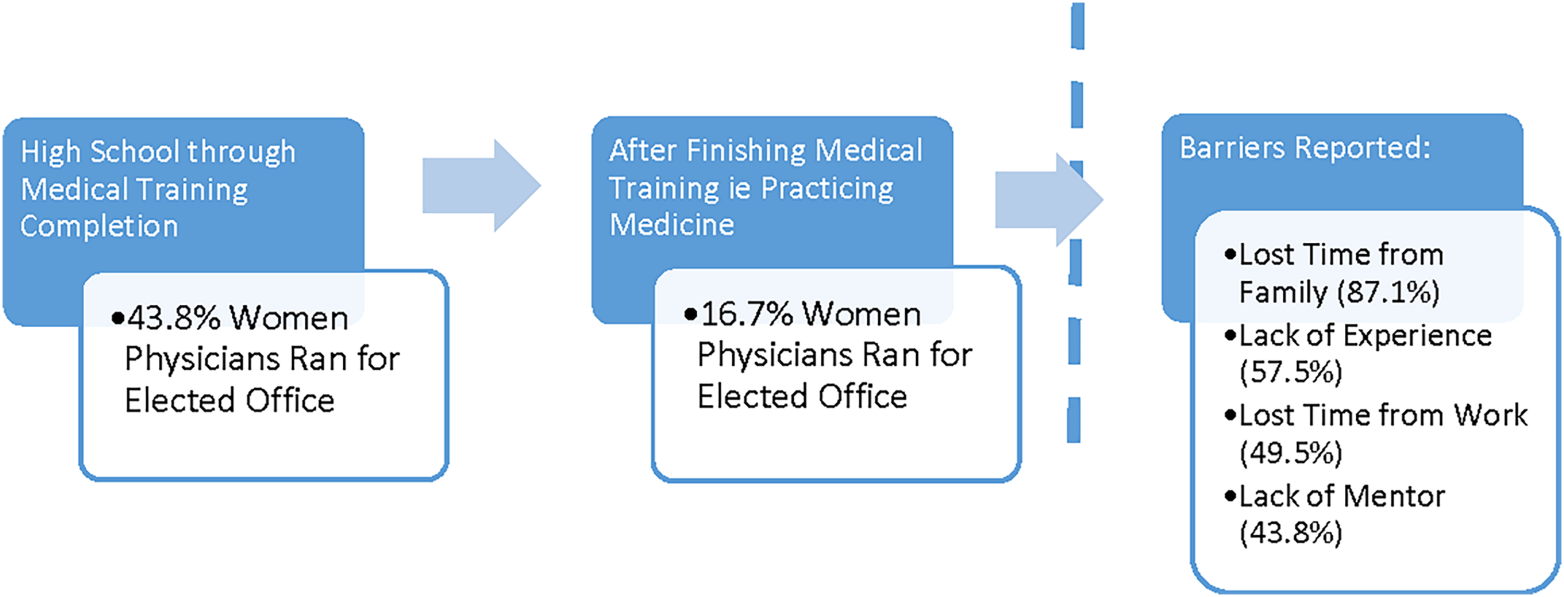
Drop-off of women physicians running for elected positions.

Our study found a decrease in the number of women physicians who ran for elected offices after completing their medical training. Although 43.8% ran for elected office before finishing residency, only 16.7% reported doing so after finishing residency (Fig. 1). Elected positions are important for physicians interested in academic promotion and also for physicians wishing to influence society and government issues in health care. Since 1960 in the U.S. Congress—some of the highest elected positions in the country—50 physicians have run for public office and only two have been women.^15^ Initial studies conducted by major medical societies demonstrate that >80% of elected offices are held by male physicians.^11^

Our study suggests that women physicians are more likely to run for elected positions during their medical training, and a significant drop-off occurs with women physicians in practice, suggesting a transition point in the career trajectory that should be further evaluated. Of interest, when we broke out the group of respondents who did report running for office after medical training, advanced age was found to be associated with running for an elected position. This finding suggests that as women advance in their careers, they are more likely to run for an elected position. A common explanation of these findings is that women with children have responsibilities that impede running for an office, yet our study did *not* find that association. This suggests there may be other contributing factors, beyond the common theory that child-rearing impedes women at all stages of their careers, to run for elected positions.

### Perceived barriers and how to overcome them

**FIG. 2.**
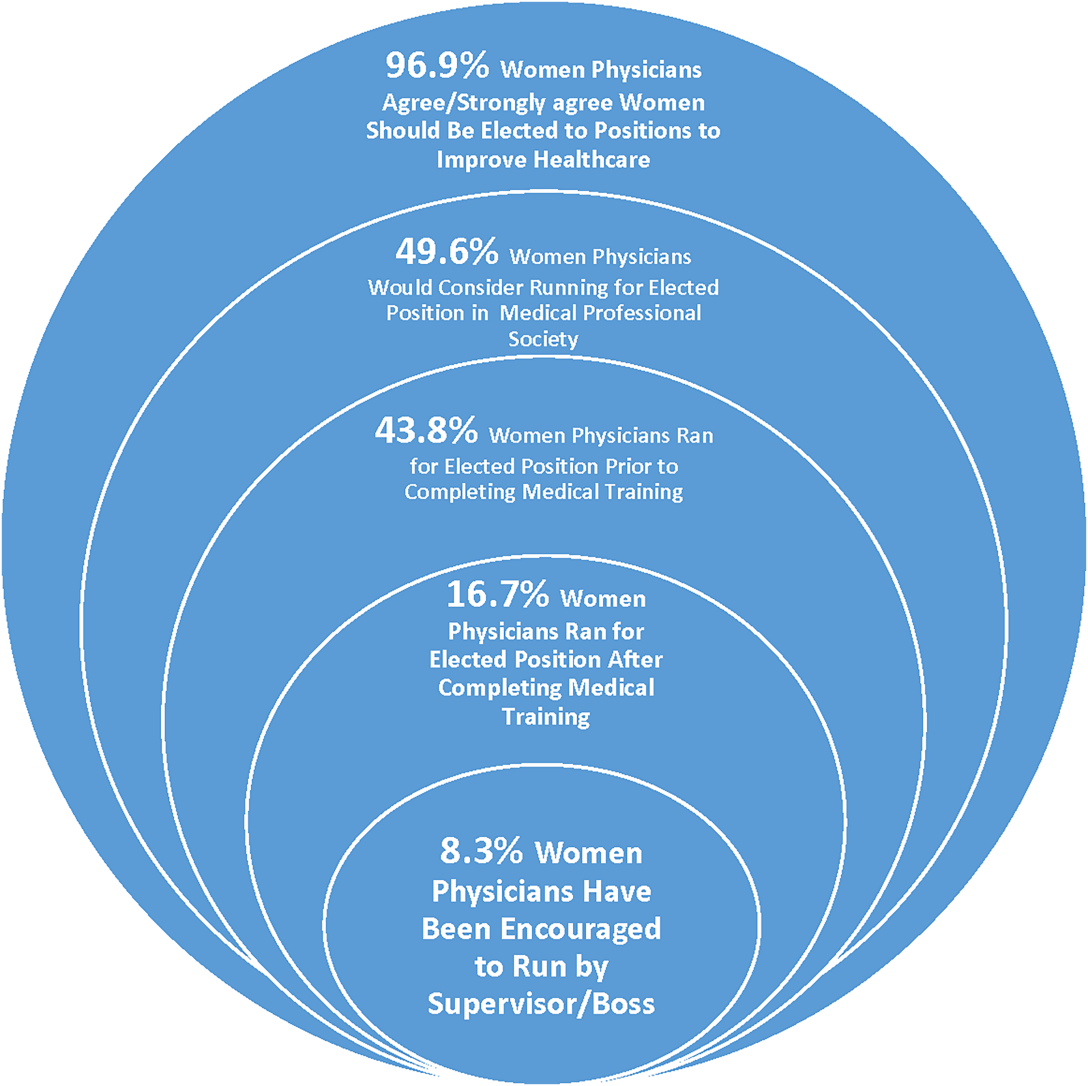
Reported data on women physicians running for elected positions in their institutions, medical/professional societies, and government.

An important part of this study was to gain an understanding of the common barriers women physicians report in their reluctance to run for elected office. Our study found that lack of protected time and work support, lack of experience, and lack of mentorship were the most common factors women physicians cite as barriers to running for elected office. In addition, fewer than 10% of the women physicians reported being encouraged to run for an elected position by their bosses or supervisors (Fig. 2). This finding is similar to previous studies demonstrating that women physicians lack sponsorship and mentorship, an important part of leadership, advancement, and promotion.^16–19^ Future studies examining the role of sponsors in supporting women physicians, and institutional support to run for elected office, may provide targeted interventions to increase the likelihood of women choosing to run.

### Limitations

Limitations of the study included the following: (1) the survey questions were given to one population, women physicians, at one point in time with no comparison group, that is, male physicians. Another limitation was that the majority of respondents were sourced from an online group of physicians who are mothers who therefore may represent a biased sample for age and motherhood. (2) As there is a scarcity of data on this topic in the literature, similar studies were not available for comparison. It is important to note that although the survey response rate was low, it is in keeping with other survey studies that used online groups of women physicians. (3) Social media was used to distribute the survey and thus may have sampled a younger subset of the physician workforce.

## Conclusion

This study revealed that women physicians are less likely to run for elected positions after completing their medical training. For those with previous election experience, the most common barriers cited were lack of institutional time and support, experience, and mentorship. Although most of the women physicians in this survey agreed that women should be in elected positions, fewer than 10% reported ever being encouraged to run by a supervisor or boss. The literature is sparse with regard to the role that elected positions have in the advancement of women in medicine, and further research is needed.

## Abbreviations Used

CI: confidence interval
OR: odds ratio
PMG: Physician Mom Group
SMD: Style M.D.
